# Can transcatheter aortic valve implantation improve cognition?

**DOI:** 10.18632/aging.102851

**Published:** 2020-02-20

**Authors:** Astrid van Nieuwkerk, Jan Baan, Ronak Delewi

**Affiliations:** 1 Amsterdam University Medical Center, University of Amsterdam, Department of Clinical and Experimental Cardiology, Amsterdam, The Netherlands

**Keywords:** aortic stenosis, transcatheter aortic valve implantation, cognition, cerebral autoregulation, cerebral outcomes

Transcatheter aortic valve implementation (TAVI) is currently the preferred treatment for patients with severe aortic valve stenosis and high surgical risk. Recent trials have found better clinical outcomes in patients with lower surgical risk scores compared to surgical aortic valve replacement. Consequently, TAVI indication is rapidly expanding to patients with intermediate and low surgical risk [1]. Prevalence of aortic valve stenosis increases with age. Due to increasing life-expectancy, the number of elderly patients with aortic valve stenosis is rising. We previously showed that nonagenarians had worse TAVI outcomes than their younger counterparts. Despite fewer comorbidities, these patients had a twofold increase in mortality, more strokes and more major or life-threatening bleeding [2]. Patients with severe aortic valve stenosis have lower cognitive scores than age, sex, and education matched individuals [3]. Especially in older patients with smaller cognitive reserves, a small change in cognition can make the difference between living independently and becoming institutionalized.

Previous studies have demonstrated that patients after TAVI have improved cognitive functioning. Khan et al. [4] conducted a meta-analysis of cognitive outcomes after TAVI of 1065 patients from eighteen studies. At one-month follow-up, overall cognition had improved in 287 patients collected from seven studies. Cognitive scores did not significantly change peri-procedurally or between baseline and longer-term follow-up up to two years.  Of all patients in this meta-analysis 21-39% had cognitive impairment, although 40% of cognitive diagnoses were missing, and definitions and cut-off scores differed. Baseline cognitive impairment was not associated with cognitive changes at any moment in follow-up, neither was stroke or embolic lesions. This finding is in contrast with prior associations between stroke and cognitive decline. Sample sizes were small with only two studies representing more than 100 patients [4]. Cognitive changes are mostly subtle and different cognitive tests measure different cognitive domains. Pooling small datasets of different cognitive test methods may therefore not result in reliable data. Looking at seven studies that used the Montreal Cognitive Assessment (MoCA) [5], that was especially designed to detect mild cognitive impairment, all studies show better test scores post-TAVI [4].

This overall improvement is interesting since TAVI can also result in cognitive decline due to cerebral (micro) infarction. Implementation of a new aortic valve releases debris and emboli from the native calcified valve. Up to 80% of TAVI patients show signs of these emboli as white matter hyperintensities on post-procedural brain imaging [6]. Most of these hyperintensities are asymptomatic and their clinical relevance remains unclear. Only 2-3% of TAVI procedures are complicated by stroke [2,4].

Schoenenberger et al. [3] found that patients with pre-existing cognitive impairment showed the greatest cognitive improvement, as well as patients with the smallest pre-TAVI aortic valve areas. This finding may be explained by restoration of blood flow to the brain. If patients have a significant aortic valve stenosis, insufficient blood can go to the brain, such that it undermines the cerebral autoregulation of brain hemodynamics. After correction of the outflow obstruction by TAVI, increased cardiac output results in increased cerebral blood supply and as a result improved cognition.

Alterations in cardiac output have different effects on young and older brains. Aging comes with a larger cerebrovascular resistance because of arteriosclerosis and sustained cerebral vasoconstriction. As a result, cerebral perfusion becomes increasingly dependent on cardiac output in the elderly [7]. TAVI enhances the outflow tract and facilitates increased cardiac output. With increased cardiac output more blood flows to the brain and consequently cerebral perfusion increases. This increased cerebral perfusion can lead to improved cerebral function and cognition. Therefore, it can be hypothesized that elderly patients benefit more from the restorative effect of TAVI. On the other hand, elderly patients are at higher risk for thromboembolic complications. This contradiction highlights the importance of informed consent and shared decision making in the elderly TAVI population.

TAVI has the ability to restore cerebral autoregulation and improve cognition, particularly in those with impaired baseline cognition or insufficient cerebral perfusion. However, it is still unknown in which patients TAVR improves cognition and in which patients TAVI may even be cognitively harmful. Its expanding indication underlines the need to further investigate cognitive outcomes in younger and healthier patients with lower surgical risk, but also in older, frail patients. This will allow us to properly select patients that will benefit not only from better cardiac function but overall quality of life.
